# Noninvasive Demonstration of Dual Coronary Artery Fistulas to Main Pulmonary Artery with 64-Slice Multidetector-Computed Tomography: A Case Report

**DOI:** 10.4061/2010/861068

**Published:** 2010-07-19

**Authors:** Yoshiki Noda, Ryo Matsutera, Yoshinori Yasuoka, Haruhiko Abe, Hidenori Adachi, Susumu Hattori, Ryo Araki, Takahiro Imanaka, Motohiro Kosugi, Tatsuya Sasaki

**Affiliations:** Cardiovascular Division, Osaka Minami Medical Center, National Hospital Organization, 2-1 Kidohigashi, Kawachinagano, Osaka 586-8521, Japan

## Abstract

Coronary artery fistulas, including coronary pulmonary fistulas, are usually discovered accidently among the adult population when undergoing invasive coronary angiographies. We report here a 58-year-old woman with dual fistulas originating from the left anterior descending coronary artery and right coronary sinus to the main pulmonary artery, demonstrating noninvasively with multidetector-computed tomography (MDCT) and transthoracic echocardiography (TTE).

## 1. Introduction

Coronary artery fistulas are rare anomalies of coronary arteries detected in around 0.1% to 0.2% of the adult population and usually discovered accidently when undergoing invasive coronary angiographies (ICA) [1, 2]. In addition, dual or multiple coronary fistulas are reported to be quite rare [3, 4]. The definite diagnoses of these patients were made only by ICA in the past. However, it has been reported that coronary artery fistulas can be detected by various kinds of noninvasive cardiac imaging, such as multidetector computed tomography (MDCT) and transthoracic echocardiography (TTE) in recent years [5, 6]. 

We report here an adult patient with dual fistulas originating from the left anterior descending coronary artery and right coronary sinus to the main pulmonary artery demonstrated noninvasively with MDCT and TTE.

## 2. Case

**Figure 1:**
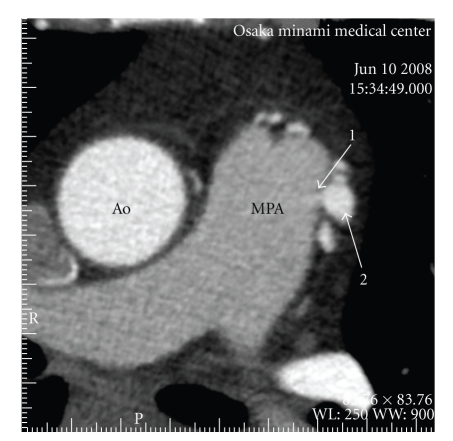
Axial images demonstrated the leakage of contrast medium (1) into the main pulmonary artery (MPA) from the aberrant artery originating from coronary arteries (2). Ao = ascending aorta.

**Figure 2:**
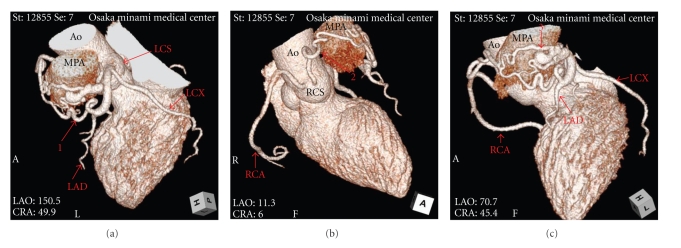
Three-dimensional volume-rendered images revealed the network of aberrant arteries arising from both left anterior descending coronary artery and right coronary sinus. Ao = ascending aorta, LAD = left anterior descending artery, LCS = left coronary sinus, LCX = left circumflex artery, MPA = main pulmonary artery, RCA = right coronary artery, RCS = right coronary sinus, 1 = the aberrant artery from left anterior descending coronary artery, 2 = the aberrant artery from right coronary sinus, 3 = the network of the aberrant arteries.

**Figure 3:**
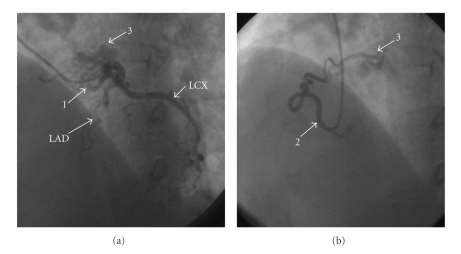
(a) The aberrant arteries from left anterior descending artery were detected with invasive coronary angiography. (b) The aberrant arteries from right coronary sinus were detected with invasive coronary angiography. LAD = left anterior descending artery, LCX = left circumflex artery, 1 = the aberrant artery from left anterior descending coronary artery, 2 = the aberrant artery from right coronary sinus, 3 = the network of the aberrant arteries.

A 58-year-old woman with no history of cardiac disease was introduced to our hospital with atypical chest pain at rest and before sleeping at night for a month. Her risk factors for coronary artery disease were obesity and dyslipidemia, and she was administered with statin by a local clinic. On clinical examination, she had no murmur, and both chest X-ray and resting electrocardiogram were normal. We performed TTE and 64-slice MDCT (Aquilion 64, Toshiba Medical Systems, Japan) since treadmill exercise test indicated positive finding for myocardial ischemia. TTE revealed continuous flow into the main pulmonary artery which had peak flow in the diastolic phase. MDCT was performed with a retrospective ECG-gated protocol and with a collimation of 64 × 0.5 mm, detector pitch of 11.2, gantry rotation time of 350 ms, tube current of 400 mA, and tube voltage of 120 kV. She received 2 mg propranolol hydrochloride and sublingual nitroglycerin before scanning, and 59 mL of contrast medium (370 mg iodine/mL) was used for MDCT angiography. Axial images demonstrated the leakage of contrast medium into the main pulmonary artery from the aberrant artery originating from coronary arteries (Figure 1), and we could not detect any other leakages of contrast medium in the pulmonary artery. In addition, three-dimensional volume-rendered images revealed the network of aberrant arteries arising from both left anterior descending coronary artery and right coronary sinus (Figure 2). From these TTE and MDCT findings, we were able to diagnose her disease as coronary to pulmonary fistulas. Furthermore, these fistulas proved to be dual fistulas originating from the left anterior descending coronary artery and right coronary sinus to the same site of the main pulmonary artery. Subsequently, ICA confirmed these fistulas (Figure 3), but we could clearly demonstrate the course and the termination of the fistulas more with MDCT. She was not referred to surgical or percutaneous treatment, because the left-to-right shunt calculated by TTE and cardiac catheterization was not significant and the absence of pulmonary hypertension, heart failure, or myocardial ischemia was detected by radionuclide myocardial perfusion imaging.

## 3. Discussion

Coronary artery fistulas are thought to be caused by either congenital or acquired factors. Acquired factors usually result from chest trauma or injury during coronary intervention or surgical procedure. The fistulas in this case were considered to be congenial, because she had no history of chest trauma or surgery. 

Most coronary artery fistulas are asymptomatic and without audible murmur. Although she had no murmur in this case, atypical chest pain appeared at rest. However, resulting from no myocardial ischemia detected by radionuclide myocardial perfusion imaging, her chest symptom was determined to be not originating from myocardial ischemia, and subsequently her symptom disappeared without therapy. 

In the past, ICA was considered to be the only definite diagnostic method of coronary anomalies, such as coronary artery fistulas. However, the reports of coronary artery fistulas detected by MDCT have increased in recent years [5–7]. It is certain that MDCT has been generally established as an accurate diagnostic modality of coronary artery disease; however; many problems, such as much radiation exposure and contrast medium still remain. With the ordinary retrospective protocol, the radiation dose of MDCT is over five times as high as that of ICA [8, 9]. Furthermore, when ICA is performed smoothly in a normal case, less contrast medium may be necessary than MDCT. However, in an anomaly case, more radiation exposure and contrast medium should be necessary because of its technical difficulties, and its dose and amount might be more than MDCT. And, we may not visualize the details of the origin, the course, and the termination of the complex fistulas due to its limited angle of angiographic projections with ICA. On the other hand, with MDCT, its three-dimensional visualization at an unlimited angle allows us to demonstrate those of the fistulas noninvasively. Furthermore, using only ICA, we may overlook the fistulas originating from coronary sinuses like this case. Therefore, MDCT is recommended for identifying coronary anomalies in a scientific statement from the American Heart Association Committee [10]. In this case, we could demonstrate the dual fistulas originating from the left anterior descending coronary artery and right coronary sinus to the same site of the main pulmonary artery by using MDCT.

Surgical or percutaneous treatment is recommended for coronary-pulmonary fistulas leading to myocardial ischemia, large left-to-right shunts, and congestive heart failure [11]. In this case, the patient was not referred to surgical or percutaneous treatment because she did not meet those criteria. Although we performed ICA, we could identify these fistulas and decide the treatment noninvasively with MDCT, TTE, and radionuclide myocardial perfusion imaging. We plan to perform the periodic noninvasive evaluation of her fistulas and, even if she has no symptom, may consider to close them when hemodynamic disorder or myocardial ischemia becomes apparent.

In conclusion, various noninvasive cardiac imagings were developed in recent years, and especially the advancement of MDCT is promising. In the future, we might be able to not only demonstrate coronary anomalies but also to make the final decision of the treatment noninvasively.
